# Death of a Distinguished Texan, Dr. Ashbel Smith

**Published:** 1886-02

**Authors:** 


Medical ★ Journal,

PUBLISHED MONTHLY AT

AUSTIN, TEXAS.

F. E. DANIEL, M. D., Editor and Publisher

Subscription Price: Two Dollars per Annum in Advance.

Papers for publication in this Journal should be in the hands of the Editor by the 10th of the month preceding the month in which it is wished to appear; they must be original,—never having appeared in print elsewhere, and must be contributed excluisvely to this Journal.

Contributions to the department of Original Articles are cordially invited from the professiom of Texas, expecially. What is most desired is Clinical reports of cases, essays and short practical articles on any medical topic; also solicited reports of proceedings of societies, clubs, etc., and items of medical news of general interest to the profession, such as notices of appointments of physicians, removals, changes, marriages, deaths, etc.

### Editorial.

### DEATH OF A DISTINGUISHED TEXAN, DR. ASHBEL SMITH.

This distinguished Scholar, Soldier, Statesman, and Physician died at his home, "Evergreen Place," on Cedar Bayou, near Houston, Texas, at 1:30 a. m., January 21, after a brief illness of pneumonia. He was in his eightieth year. His remains were brought to Austin under military escort, attended by a delegation of citizens and physicians, and laid in state at the Capitol till 2 p. m. Sunday, the 24th. At that hour Bishop Elliott, of the Episcopal Church, delivered a most impressive extempore eulogy on the life, services and character of the deceased; after which the funeral procession, headed by the military companies of Houston and Austin, moved to the State Cemetery, and all that was mortal of this good and great man was laid away with the ashes of many of Texas' departed great sons. There was a general outpouring of people to do the last sad honors to the memory of Dr. Smith,—and the funeral procession was the largest ever seen in Austin.

The following brief outline of his life is taken from the *Houston Daily News:*

THE LATE DR. ASHBEL SMITH.

In the death of Dr. Ashbel Smith, at his home in Harris county, yesterday, Texas has lost one of the most useful, estimable, gifted and cultured of her veteran citizens, medical science and general scholarship one of their distinguished ornaments, and the world at large a man of rare force, and singular completeness of self-poised individuality. Ashbel Smith was born at Hartford, Conn., in 1806, and graduated at Yale College in 1824. He began the study, and finally graduated in medicine. When a young man, he emigrated to North Carolina, and afterward went to the city of Paris in order to perfect himself in the study and practice of medicine and surgery. He came to Texas in 1837, and was soon appointed Surgeon-General of the Republic. On retiring from that position, he came to Galveston to enter upon the practice of his profession, and was one of the physicians in the first yellow fever epidemic there, that of 1839. Neither he nor any of the other physicians there had ever before seen the disease, and all regretted their lack of experience in it, and their inability to effect cures; but their success was about equal to that of those most familiar with that intractable disease. The deceased was somewhat discouraged by his experience, though he wrote a pamphlet on the epidemic, and described faithfully the practice and results. In 1842, President Houston appointed him to the office of Minister from Texas to France and Great Britain, a position which he filled with ability and honor. He continued to represent Texas at the courts of France and Great Britain until the young Republic was annexed to the United States. On his return, he accompanied the United States Army under General Taylor into Mexico. In 1852, and again in 1855, he was one of the United States Commissioners, and presided over the Board to visit and report upon the condition of the Military Institute at West Point. He was a member of the Legislature for Harris county for several terms, both before and after the late civil war. He was among the first to volunteer for the Confederate service in 1861, and raised a company at Bayland, his home, on Galveston Bay. He was promoted to the command of the Second Regiment of Texas Infantry, served throughout the war, and was in command of the Port of Galveston on the final surrender, in June, 1865. In 1878, he represented Texas as Commissioner to the World's Exposition at Paris. His last election to the Legislature was in 1879, for the succeeding two years. [Dr. Smith was President of the Texas State Medical Association in 1881.] His only public service since has been as one of the Regents of the State University. The deceased was a man of much learning, and of large experience in public affairs and intercourse with the world. His reading was extensive and varied—historical, scientific, literary and political. His manner was polite, and his language always precise and polished. He never spoke without the use of language correct and graceful enough for the forum or the press. He never married, and his domestic habits were frugal and simple. He had no taste for pageantry or display, but found his chief pleasures in books, and the society of men of learning and intelligence. He had entered upon the eightieth year of his age, and his regular life, cheerful temper and congenial pursuits had preserved his bodily and intellectual vigor to a remarkable degree.

